# Prevalence of Coaddictions and Rate of Successful Treatment Among a French Sample of Opioid-Dependent Patients With Long-Term Opioid Substitution Therapy: The OPAL Study

**DOI:** 10.3389/fpsyt.2019.00726

**Published:** 2019-10-17

**Authors:** Marie Grall-Bronnec, Edouard-Jules Laforgue, Gaëlle Challet-Bouju, Jennyfer Cholet, Jean-Benoit Hardouin, Juliette Leboucher, Morgane Guillou-Landréat, Caroline Victorri-Vigneau

**Affiliations:** ^1^Addictive Medicine and Psychiatry Department, CHU Nantes, Nantes, France; ^2^INSERM UMR 1246, SPHERE, Methods in Patient-Centered Outcomes and Health Research, Nantes and Tours University, Nantes, France; ^3^HUGOPSY Network, Nantes, France; ^4^Clinical Pharmacology Department, CHU Nantes, Nantes, France; ^5^Methodology and Biostatistic Department, DRCI, CHU Nantes, Nantes, France; ^6^Addictive Medicine Department, CHU Brest, Brest, France; ^7^Université de Bretagne Occidentale, ERCR SPURBO, Brest, France

**Keywords:** opioid use disorder, opioid substitution therapy, opioid use disorder treatment, coaddictions, problem gambling, unsuccessful treatment

## Abstract

**Background:** Opioid use disorder (OUD) is a worldwide major health concern due to increased early mortality and morbidity. Opioid substitution therapy (OST) is approved in the context of a global OUD treatment (OUDT), in conjunction with psychosocial interventions. Many factors can explain why unsuccessful treatment rates remain high. While the phenomenon of addiction switching is often proposed, it is not known whether this also includes gambling addiction. The primary objective of the OPAL study was to determine the prevalence of coaddictions, including problem gambling, among patients with OUDT. Secondary objectives were to assess the rate of unsuccessful OUDT and to characterize the associated factors.

**Methods:** For this observational transversal multicenter study, patients with OUDT including OST for at least 6 months were recruited. Clinical assessment was based on a clinically structured interview and a set of self-reported questionnaires. Coaddictions were screened using the Fagerström, the CRAFFT, and the Lie/Bet questionnaires. Unsuccessful OUDT was defined as the persistence of opioid use and/or the worsening of another substance use or gambling practice. After a descriptive analysis, a multivariate analysis was performed to identify the factors associated with unsuccessful OUDT.

**Results:** The sample consisted of 263 patients. Prevalence of coaddictions reached 97% of the sample. Problem gambling was associated with 10% of the patients. OUDT was considered as “unsuccessful” for 60% of the patients. Associated factors included having drug-using friends, psychiatric and professional negative consequences related to opioid use, more than one OST-prescribing physician, and impulsivity, especially high scores for lack of premeditation and sensation seeking.

**Conclusions:** This study provides further evidence of the need to consider coaddictions and the usefulness of global addictive evaluations. Poor prognostic factors must alert the clinician to initiate more sustained care. Further implications are discussed.

## Introduction

Over the past decade, medical and nonmedical opioid use has soared dramatically (1). Resurgence of heroin use in some countries, such as the United States, and misuse of pharmaceutical opioids and illicit fentanyl derivatives are some of the reasons for the current “opioid crisis phenomenon” (2, 3). In 2018, the worldwide prevalence of opioid use disorder (OUD) was estimated at 0.7% for individuals aged 15 to 64 years (3). Opioid use is associated with increased early mortality and morbidity. Due to a dramatic increase in lethal overdose rates (4), OUD is a major, and increasing, public health concern. Moreover, due to medical complications, such as infections, and frequently associated socioprofessional issues, the burden of harm is estimated by the World Health Organization at 9.2 and 11.2 million disability-adjusted life-years for opioid use and opioid dependence, respectively (5, 6).

OUD is associated with strong withdrawal symptoms that are caused by cessation or reduction of opioid use and craving, both of which are responsible for continual use and unsuccessful attempts to stop consumption despite their awareness of the negative consequences. OUD treatment (OUDT) includes psychosocial treatment combined with a medication. The choice among the available treatment options should be a shared decision between the clinician and the patient. In France, two medications are available as opioid substitution therapy (OST): buprenorphine and methadone. Buprenorphine, which is a partial mu agonist, can be prescribed by any physician. Methadone, which is a full mu agonist, is less accessible, since it is a listed narcotic. Its prescription is initially restricted to physicians operating in specialized units or hospitals. After a period of stabilization, follow-up and prescription may be carried out by any physician. Methadone and buprenorphine are both approved as pharmacological treatments in the context of OUDT, in order to 1) suppress opioid withdrawal, 2) block the effects of illicit opioids, 3) reduce opioid craving and stop or reduce the use of illicit opioids and prevent relapse, 4) stop drug injection to reduce the spread of blood-borne viruses, and 5) promote and facilitate patient engagement in recovery-oriented activities including psychosocial intervention (7–9). Evidence suggests that the use of these medications is superior to other treatment options for most patients with OUD and that methadone and buprenorphine are equally effective (10, 11). In conjunction with pharmacotherapy, psychosocial treatment includes behavioral interventions by qualified healthcare providers in order to assess psychosocial needs, provide supportive counseling, and promote existing family support links and referral to community services (9).

Assessing the success of OUDT is challenging. The main issue resides in the selection of relevant outcome measures. From the perspective of population health, outcomes could be overdose mortality rate (12), prevalence of HIV (13) and hepatitis C virus infection acquisition (14), or cessation of injection drug use (15). From the perspective of the individuals health, controlled trials often assess the outcome as the time to opioid relapse (16), retention in treatment (17), self-reported craving and opioid withdrawal symptoms (18), opioid-positive urine drug tests (10), psychological and social well-being (19), or quality of life (20). However, there is no consensus as to which could be the most appropriate outcome measure. This is compounded by the difficulty in performing long-term studies with “real life” patients, i.e., complex patients with both somatic, psychiatric, and addictive comorbidities and social vulnerabilities. In particular, comorbid substance use disorders are frequent conditions among patients with OUD, as they may develop, persist, or worsen during or after the OUDT (21–24). Studies measuring the propensity of patients with OUDT to resort to abuse of other substances are inconclusive; the published results report increased, decreased, or unchanged use (25–27). The classic situation, however, is “switching addiction,” or the increased consumption over time of other substances. The scenario may be that OUDT is associated with clinical improvement and the cessation of opioid use; however, the onset or aggravation of other addictive disorders, such as alcohol or another substance use disorder, is often observed (25, 28, 29). Behavioral addictions seem to be less reported. Gambling disorder is the only disorder classified as a behavioral addiction in the *Diagnostic and Statistical Manual of Mental Disorders, Fifth Edition* (*DSM-5*) (30). Despite the fact that it is known that patients treated for substance use disorders are at higher risk of gambling disorder than the general population (31–33), scientific studies on the switch from OUD to gambling disorder are scarce.

To summarize, patients with OUD could be effectively treated regarding their use of opioids, but their long-term evolution could be hampered with comorbid addictions, mitigating their global improvement. The primary aim of our study was to determine the prevalence of coaddictions, including gambling disorder, in a large sample of patients treated for an OUD. Secondary objectives were to assess the rate of unsuccessful OUDT and to characterize the associated factors.

## Materials and Methods

### Procedure and Ethics

OPAL (NCT01847729) was an observational, cross-sectional, multicenter study involving 10 centers located in the Western region of France. It combined both a clinical evaluation and a prespecified ancillary pharmacogenetic study (34). OPAL was conducted in accordance with the Good Clinical Practice Guidelines and the Declaration of Helsinki and was approved by the local ethics committee. Written informed consent was collected from all participants.

### Participants

The study included patients 18 years or older, receiving OST (methadone or buprenorphine) for at least 6 months for opiate dependence (according to the *DSM-IV*) (35). The period of 6 months was chosen since it is the time generally necessary to adapt and stabilize the dosage of the OST in the context of a global OUDT. Exclusion criteria were difficulties reading or writing in French and guardianship.

### Measures

The clinical evaluation consisted of a hetero-assessment (structured interview conducted by one of the investigators) and a self-assessment (self-answered questionnaires completed by the patient). The following data were collected:

#### Sociodemographic Characteristics

We collected data including sex, age, educational level, marital and parental status, housing, social support, professional status, and financial situation.

#### Impulsivity Characteristics

Attention-deficit/hyperactivity disorder (ADHD) was assessed by two self-report questionnaires: the Wender Utah Rating Scale–Child (WURS-C) (36) to make a retrospective screening of ADHD in childhood and the Adult ADHD Self-report Scale Symptom Checklist (ASRS v1.1) (37), which screens ADHD in adulthood. The WURS-C specificity is satisfactory, which limits the risk of a wrong diagnosis. It is designed to assess ADHD symptoms represented by 25 items on 5-point Likert scales. This ASRS v1.1 is based on the 18 diagnostic criteria of the *DSM-IV, Text Revision*, scored according to their frequency. Some authors have concluded that the ASRS v1.1 screen is a simple screening tool that is useful and has an acceptable validity for the identification of ADHD among addicted patients. In particular, the sensitivity and negative predictive value are acceptable, as is required of a screening tool. Based on the results on these questionnaires, it was possible to screen ADHD in childhood (WURS-C score ≥46/100) and ADHD probably persistent at adulthood (WURS-C score ≥46/100 and ≥4/6 marks in the dark shaded boxes within part A of the ASRS v1.1). Impulsivity was scored using the short version of the UPPS Impulsive Behavior Scale (short UPPS-P) (38). The UPPS-P scale is a self-report questionnaire that interrogates five complementary impulsivity constructs: positive urgency, negative urgency, lack of perseverance, lack of premeditation, and sensation seeking. The short UPPS-P is a 20-item version reduced from the initial 59-item UPPS-P.

#### OUD Characteristics

The evaluation was performed on a single time point, but the patients were questioned on two distinct periods: before the OST initiation and since the OST initiation.

Before the OST initiation. We collected data including the age at first opioid experimentation, age at opioid dependence, age at the first attempt to stop opioid use (withdrawal or OST), main substance used (heroin, nonmedical use of codeine, morphine, buprenorphine, and methadone), main route of administration (nasal, intravenous, inhaled, oral), and negative consequences related to opioid dependence (financial, socioaffective, psychiatric, professional, legal, physical problems).

Since the OST initiation. We collected the characteristics of OST (type of medication, duration, OST initiation with daily supervised dosing by a qualified health professional, maximal daily dose, current daily dose, compliance, withdrawal symptoms, current opioid use despite OST or opioid abstinence, defined as the self-reported absence of opioid use over the previous 6 months). The 6-month period was chosen because it corresponds to the time usually required to stabilize the treatment dosage and because each patient could be asked about this period.

#### Use of Other Substances and Gambling Habits

Patients were asked about the current frequency of substance use [nicotine, alcohol (>3 standard units *per* day for the men and >2 for the women), cannabis, cocaine, amphetamines, lysergic acid diethylamide (LSD) or other synthetic drugs, benzodiazepines, or barbiturates] and gambling practice. Coaddictions were screened using the Fagerström nicotine dependence test, the CRAFFT (for Car, Relax, Alone, Forget, Family/Friends, Trouble) questionnaire, and the Lie/Bet questionnaire. The Fagerström test is a noninvasive and easy-to-obtain self-report tool that conceptualizes dependence through physiological and behavioral symptoms (moderate to high nicotine dependence if score ≥2/7) (39). The CRAFFT questionnaire is a powerful tool for the identification of alcohol and substance misuse among adolescent and young adult population. Two risk levels were thus defined: a moderate risk (score >2/6), largely identifying regular consumption of alcohol and substance, and a high risk (score >3/6), identifying the severity of consumption (40). The Lie/Bet questionnaire was used to differentiate problem and nonproblem gamblers. It was based on the following two criteria from the *DSM-IV*: “Have you ever had to lie to people important to you about how much you gambled?” (Lie) and “Have you ever felt the need to bet more and more money?” (Bet). We concluded that gambling was potentially problematic (currently or in the past) when at least one of the replies was positive. We chose this questionnaire because It has excellent sensitivity, which is crucial for a screening instrument, and because its specificity is also very good, (41). Patients were asked about the progression of their substance use or gambling habits since OST initiation (stability/improvement/worsening).

#### Outcome Measures

The primary outcome measure in this study was the presence of at least a coaddiction, defined by a moderate/high nicotine dependence and/or a high-risk substance use [alcohol (>2 or 3 standard units *per* day), cannabis, cocaine, amphetamines, LSD or other synthetic drugs, benzodiazepines, and/or barbiturates] and/or a problem gambling. The secondary outcome measure was the OUDT status: “successful OUDT” was defined by opioid abstinence (see above) and the stability or improvement of other substance use or gambling practice, and “unsuccessful OUDT” by the persistence of opioid use and/or the worsening of another substance use or gambling practice.

### Statistical Analysis

A descriptive statistical analysis of the sociodemographic and clinical characteristics was conducted for the entire sample. Continuous variables were described by the mean and standard deviations, and categorical variables were presented by numbers and percentages.

We divided the sample into two groups according to status at inclusion (“successful OUDT” and “unsuccessful OUDT”) and then compared these groups. Bivariate analyses were conducted to explore the associations between the status at inclusion and the set of variables mentioned above. *χ* ^2^ Tests or Fisher tests were used to analyze the qualitative variables, and Student or Wilcoxon tests for quantitative variables.

Thereafter, multivariate analyses were performed using an iterative selection procedure to select the variables that were significantly associated with the “unsuccessful OUDT” status, as assessed by the likelihood ratio test (variable candidates for the model were those associated with unsuccessful OUDT in bivariate analyses with the *P* < 0.20 criterion and subsequently selected in the model using the *P* < 0.05 criterion). The corresponding odds ratio and associated 95% confidence interval were estimated. The ability of the final logistic model to discriminate between successful or unsuccessful OUDT was assessed using the area under the receiver operating characteristic (ROC) curve, and the goodness of fit of the model was assessed using the Hosmer–Lemeshow test. The statistical analyses were carried out with SAS 9.1 and R statistical software (SAS Institute, Inc.).

## Results

A total of 263 patients were included in the study.

### Description of the Sample

#### Sociodemographic Characteristics

As shown in **Table 1**, 75% of the sample were men. The majority had an education level lower than year 12 and were unemployed. Almost all participants had stable housing, and about a third lived in a relationship. Almost all participants declared having social support. However, three-quarters had close friends who were drug users.

**Table 1:** Sociodemographic and impulsivity characteristics of the sample (n = 263).

	% of Mean (SD)
**Sociodemographic Characteristics**	
**Sex** (% males)	75%
**Age** (y)	34.9 (7.4)
**Living conditions**	
Marital status (% living as a couple)	39%
Drug-using spouse	25%
≥1 Dependent child	47%
Stable housing	89%
Social support	91%
Drug-using friends	95%
**Educational attainment** (% > 12 y)	13%
**Work status and financial situation**	46%
Social benefits	35%
No income	5%
Debt	36%
**Impulsivity Characteristics**	
**ADHD (n = 222)**	
ADHD in childhood (WURS-C)	44%
ADHD persistent in adulthood (ASRS)	29%
**Impulsivity (UPPS-P) (n = 238)**	
Negative urgency (/16)	10.5 (2.9)
Positive urgency (/16)	10.4 (2.6)
Lack of premeditation (/16)	8.4 (2.3)
Lack of perseverance (/16)	8.4 (2.6)
Sensation seeking (/16)A	10.7 (2.9)

ADHD, attention-deficit/hyperactivity disorder.

#### Impulsivity Characteristics

The profile of the patients is marked by impulsivity, as shown in **Table 1**. The average scores on the UPPS-P questionnaire were high for the five dimensions, but especially for the Urgency (positive and negative) and Sensation Seeking dimensions. In addition, almost half of the sample presented characteristics of ADHD in childhood, and ADHD probably persisted into adulthood for almost a third of the patients.

#### Opioid Use Disorder Characteristics

Before the OST initiation. The main stages (first experimentation, onset of dependence, first attempt to stop) of the opioid use trajectory followed each other at a rapid pace (**Table 2**). The vast majority (90%) of patients reported consuming mainly heroin, and the main route of opioid administration was the nasal route. Patients reported a number of negative consequences related to their opioid use, mainly financial (71%), socioaffective (70%), and psychiatric (69%).

**Table 2:** Opioid use disorder characteristics of the sample (n = 263).

	% or Mean (SD)
**Before OST Initiation**	
**Stages of OUD (age, y)**	
First experimentation	20.4 (5.1)
Onset of dependence	22.9 (5.7)
First attempt to stop	26.0 (6.0)
**Opioid mainly used**	
Heroin	90%
Codeine	4%
Buprenorphine	3%
Morphine	2%
Varying	1%
**Main route of administration**	
Nasal	60%
Intravenous	23%
Inhaled	11%
Oral	4%
Varying	1%
**OUD negative consequences**	
Financial	71%
Socioaffective	70%
Psychiatric	69%
Professional	55%
Judicial	48%
Somatic	33%
**Type of first attempt to stop**	
OST initiation	57%
Withdrawal	42%
**Since OST Initiation**	
**Current OST**	
Methadone	68%
Buprenorphine	32%
** OST initiation with daily supervised dosing**	80%
** Current daily dose (mg/day)**	
Methadone	57.4 (32.7)
Buprenorphine	7.4 (5.5)
**Maximal daily dose (mg/day)**	
Methadone	84.6 (36.3)
Buprenorphine	11.0 (5.7)
**OST length (mo)**	51.0 (4.3)
**Poor compliance**	7%
**Presence of opioid withdrawal symptoms**	17%
**Current opioid abstinence (excluding OST)**	75%

OST, opioid substitution therapy; OUD, opioid use disorder.

Since the OST initiation. At the time of inclusion in the study, the prescribed OST was methadone for two-thirds of patients, with average treatment duration of 51 months (**Table 2**). For most of them, OST was initiated with daily supervised dosing by a qualified health professional. OST compliance was rated as good by the clinician for the vast majority of patients. However, 17% reported signs of opioid withdrawal. A quarter of patients still reported opioid use.

### Prevalence of Coaddictions

Two hundred forty of 263 patients completed self-report questionnaires that allowed current coaddiction assessment. Current substances misuse and problem gambling are described in **Table 3**. Nicotine dependence was present among more than three-quarters of the responders. Substance use other than nicotine was characterized as “high risk” for the majority of the responders [212 patients (88%)]. Substances mainly misused were alcohol [166 patients (70%)], cannabis [156 patients (65%)], and cocaine [107 patients (45%)]. Gambling was problematic for 23 patients (10% of the responders). In total, 232 of 240 patients (97%) were identified with nicotine dependence and/or high risk for other substance use and/or problem gambling.

**Table 3:** Prevalence of current coaddictions (n = 240).

	%
**Nicotine** (Fagerström test ≥2)	76%
**Other substances** (CRAFFT screening test ≥2)	
At least one substance	88%
Alcohol (>2 or 3 standard units per day)	69%
Cannabis	65%
Cocaine	45%
Amphetamines	22%
LSD or other synthetic drugs	20%
Benzodiazepines or barbiturates	30%
**Gambling** (Lie/Bet questionnaire ≥1)	10%

LSD, lysergic acid diethylamide.

### Evolution of Substance Use and Gambling Since OST Initiation

As previously mentioned, 63 of 252 patients (25%) still reported opioid use. Moreover, substance use (including nicotine) or gambling worsened for 112 of 240 patients (47%) since OST initiation. Results are presented in **Table 4**.

**Table 4:** Evolution of substance use and gambling since OST initiation (n = 240).

	Stability	Improvement	Worsening
		Number of patients (%)	
Nicotine	158 (65%)	34 (14%)	48 (20%)
Alcohol (>2 or 3 standard units *per* day)	144 (60%)	39 (16%)	57 (24%)
Cannabis	175 (73%)	50 (21%)	15 (6%)
Cocaine	164 (68%)	58 (24%)	18 (8%)
Amphetamine	203 (85%)	34 (14%)	3 (1%)
LSD or other synthetic drugs	205 (85%)	33 (14%)	2 (1%)
Benzodiazepines or barbiturates	192 (20%)	24 (10%)	24 (10%)
Gambling	228 (95%)	7 (3%)	5 (2%)

LSD, lysergic acid diethylamide; OST, opioid substitution therapy.

### Rate of Successful/Unsuccessful OUDT

Of the 23 patients who did not return their self-report questionnaires, 12 could be classified in the “unsuccessful OUDT” group as they reported continuing opioid use during the structured interview. We were therefore able to determine the OUDT status for 252 patients. OUDT was considered as “successful” for 101 (40%) and as “unsuccessful” for 151 (60%) of them. Conditions of “unsuccessful OUDT” are shown in **Table 5**.

**Table 5:** Description of the OUDT status (n = 252).

	Number of patients (%)
**Successful OUDT**	
**Opioid abstinence without worsening of other substance use or gambling**	**101 (40%)**
**Unsuccessful OUDT**	
**Persistence of opioid use without worsening of other substance use or gambling**	**27 (11%)**
**Persistence of opioid use with worsening of other substance use or gambling**	**26 (10%)**
Nicotine	8
Alcohol (>2 or 3 standard units *per* day)	11
Cannabis	4
Cocaine	5
Amphetamines	2
Benzodiazepines or barbiturates	5
Gambling	1
**Opioid abstinence but worsening of other substance use or gambling**	**84 (33%)**
Nicotine	37
Alcohol (> 2 or 3 standard units *per* day)	46
Cannabis	10
Cocaine	13
Amphetamines	1
LSD or other synthetic drugs	1
Benzodiazepines or barbiturates	19
Gambling	4
**Persistence of opioid use with missing data about other substance use or gambling**	**12 (5%)**
**Missing data about opioid use but worsening of other substance use or gambling**	**2 (1%)**
Cannabis	1
LSD or other synthetic drugs	1

LSD, lysergic acid diethylamide; OUDT, opioid use disorder treatment.

### Factors Associated With Unsuccessful Oudt

Characteristics related to sociodemographics, impulsivity, OUD, and other substance use or gambling were compared between the two groups of patients. Results are presented in **Table 6**.

**Table 6:** Comparison of patients with successful or unsuccessful OUDT (n = 252).

	Successful OUDT(n = 101)	Unsuccessful OUDT(n = 151)	
	Number of patients (%) or mean (SD)		*P*
**Sociodemographic Characteristics**			
**Sex (% males)**	72 (71%)	116 (77%)	0.32
**Age (y)**	35.5 (7.6)	34.2 (7.1)	0.17
**Living conditions**			
Marital status (% living as a couple)	45 (45%)	48 (32%)	**0.048**
Drug-using spouse	20 (20%)	25 (17%)	0.15
≥1 Dependent child	32 (32%)	36 (24%)	0.17
Stable housing	94 (93%)	128 (85%)	0.10
Social support	96 (95%)	134 (89%)	0.082
Drug-using friends	66 (65%)	119 (79%)	0.018
**Educational attainment (>12 y)**	13 (13%)	20 (13%)	0.92
**Work status and financial situation**			
Employed workers	55 (54%)	63 (42%)	0.042
No income	2 (2%)	10 (7%)	0.13
Debt	32 (32%)	59 (39%)	0.23
**Before OST Initiation**			
**Stages of OUD (age, y)**			
First experimentation	21.3 (5.5)	19.8 (4.8)	**0.0275**
Onset of dependence	23.7 (5.8)	22.1 (5.3)	**0.0256**
First attempt to stop	26.6 (6.2) (n = 100)	25.4 (5.5) (n = 148)	0.11
**Opioid mainly used**			
Heroin	88 (87%)	139 (92%)	0.35
Codeine	5 (5%)	4 (3%)	
Buprenorphine	4 (4%)	4 (3%)	
Morphine	4 (4%)	2 (1%)	
Varying		2 (1%)	
**Main route of administration**			0.46
Nasal	58 (58%)	91 (61%)	
Intravenous	25 (25%)	35 (23%)	
Inhaled	10 (10%)	19 (13%)	
Oral	6 (6%)	4 (3%)	
Varying	1 (1%)	2 (1%)	
**Daily use**	94 (93%)	145 (97%)	0.19
**OUD negative consequences**			
Psychiatric	61 (60%)	116 (77%)	**0.005**
Somatic	36 (36%)	46 (30%)	0.39
Professional	48 (48%)	91 (60%)	**0.046**
Socioaffective	69 (68%)	108 (72%)	0.59
Judicial	43 (43%)	81 (54%)	0.085
Financial	77 (76%)	101 (67%)	0.11
**Type of first attempt to stop**			
OST initiation	53 (53%)	92 (62%)	0.13
Withdrawal	48 (48%)	56 (38%)	
**Since OST Initiation**			
**Current OST**			**0.006**
Methadone	58 (57%)	123 (75%)	
Buprenorphine	43 (43%)	37 (25%)	
**OST initiation with daily supervised dosing**	(76) 75%	126 (85%)	0.066
**“Nomadism”**			
Multiple prescribing physicians	5 (5%)	19 (13%)	**0.038**
Multiple dispensing pharmacies	8 (8%)	15 (10%)	0.55
**OST length (mo)**	51.9 (57.1)	49.7 (48.2)	0.74
**Current daily dose (mg/day)**			
Methadone	48.2 (31.1) (n = 58)	61.1 (31.7) (n = 113)	**0.0121**
Buprenorphine	6.9 (4.5) (n = 41)	7.4 (5.7) (n = 32)	0.65
**Maximal daily dose (mg/day)**			
Methadone	79.5 (35.9) (n = 57)	86.7 (35.7) (n = 113)	0.29
Buprenorphine	10.5 (5.3) (n = 41)	11.7 (5.6) (n = 31)	0.37
**Presence of opioid withdrawal symptoms**	11 (11%)	34 (23%)	0.018
**Poor compliance**	2 (2%)	13 (9%)	**0.028**
**Current opioid abstinence**	101 (100%)	84 (56%)	**<0.001**
**Impulsivity Characteristics**			
**ADHD**			
ADHD in childhood (WURS-C)	37 (40%) (n = 93)	61 (47%) (n = 129)	0.27
ADHD persistent in adulthood (ASRS)	19 (21%) (n = 92)	35 (27%) (n = 129)	0.22
**Impulsivity (UPPS-P)**			
Urgency (/16)	10.1 (2.7) (n = 98)	10.9 (3.0) (n = 137)	**0.0365**
Positive urgency (/16)	9.7 (2.7) (n = 96)	10.8 (2.4) (n = 138)	**0.0011**
Lack of premeditation (/16)	7.9 (2.3) (n = 96)	8.7 (2.2) (n = 134)	**0.0068**
Lack of perseverance (/16)	8.0 (2.7) (n = 97)	8.7 (2.5) (n = 121)	**0.0371**
Sensation seeking (/16)	9.9 (2.9) (n = 99)	11.2 (2.8) (n = 139)	**0.0005**
**Current Coaddictions**			
**Nicotine (Fagerström test ≥2)**	68 (67%)	115 (76%)	**0.12**
**Other substances (CRAFFT substance abuse screening test ≥2)**			
At least one substance	82 (81%)	130 (86%)	**0.30**
Alcohol (>2 or 3 standard units per day)	62 (61%)	104 (69%)	**0.22**
Cannabis	60 (59%)	96 (64%)	**0.50**
Cocaine	30 (30%)	77 (51%)	**0.001**
Amphetamines	15 (15%)	38 (25%)	**0.049**
LSD	16 (16%)	32 (21%)	**0.29**
Benzodiazepines or barbiturates	18 (18%)	53 (35%)	**0.003**
**Gambling (Lie/Bet questionnaire ≥1)**	5 (5%)	18 (12%)	**0.060**

OST, opioid substitution therapy; OUDT, opioid use disorder treatment.

**Table 7** shows the results for the multivariate analysis of the “unsuccessful OUDT” model [only the significant variables (*P* < 0.05) at the end of the descending procedure are shown]. After excluding observations with missing data, 238 patients were included in the analysis. In our model, we observed factors associated with unsuccessful OUDT: having drug-using friends, having psychiatric and professional negative consequences related to opioid use, having more than one OST-prescribing physician, and having high scores for the UPPS-P lack of Premeditation and Sensation Seeking scales. In contrast, living as a couple and having somatic and financial negative consequences related to opioid use could be considered as protective factors.

**Table 7:** Factors associated with unsuccessful OUDT—multivariate logistic regression model (n = 238).

Variables	Adjusted OR	CI_95%_ (OR)	*P*
Living condition (in couple)	0.39	[0.20; 0.73]	0.004
Drug-using friends	2.03	[1.04; 3.97]	0.037
Psychiatric negative consequences	3.66	[1.81; 7.40]	<.001
Somatic negative consequences	0.45	[0.23; 0.87]	0.018
Professional negative consequences	1.97	[1.05; 3.69]	0.035
Financial negative consequences	0.29	[0.14; 0.60]	0.001
Multiple prescription physicians	7.06	[2.04; 24.38]	0.002
Lack of premeditation	1.20	[1.45; 1.38]	0.011
Sensations seeking	1.23	[1.10; 1.38]	<.001

CI, confidence interval; OR, odds ratio; OUDT, opioid use disorder treatment.

The Hosmer–Lemeshow goodness-of-fit test showed that the final model was well calibrated with *P* = 0.33 (a large *P* value indicating a good model fit), and the area under the ROC curve was 0.79, showing that the model discriminated well between patients with “unsuccessful OUDT” and patients with “successful OUDT.”

## Discussion

### Main Results

In the present study, we estimated the prevalence of coaddictions, including problem gambling, and the rate of unsuccessful treatment in a large sample of patients with OUD. To the best of our knowledge, this is the first study to explore this topic in such a broad manner. First, we demonstrated that almost all patients who completed the self-report questionnaires could be considered as having at least another current coaddiction (nicotine dependence and/or high risk for other substances use and/or problem gambling). This observation should prompt us to consider the patient’s situation as a whole and not to focus only on OUD, even if it is the main disorder and the reason for referral. Furthermore, patients reported that substance use or gambling substantially worsened, especially considering nicotine and alcohol use. Pursuit of former substances use disorder, especially for alcohol, is not uncommon and has previously been described (25, 28, 29, 41). The “switching addiction” phenomenon could explain this aggravation. Addictive disorders share an underlying biopsychological process, resulting in an interaction of impairments of motivation-reward, affect regulation, and behavioral inhibition (42). This is all the more worrying as alcohol use is known to promote illicit drug relapse (23, 41). Alcohol acts on the opioid system, which could explain the propensity of opioid users to use alcohol when they quit opioids. A study showed that high-dose buprenorphine could be of superior effectiveness compared to methadone in alcohol use reduction (43). Our study was not designed to compare the relative efficacy of methadone and buprenorphine, but it seems that buprenorphine is more beneficial for concomitant alcohol use disorder.

Second, it is interesting to note that some patients reported that their substance use decreased, although reduction in other substance use is not an explicit goal of OUDT. Therefore, this represents a “secondary benefit” of OUDT that supports the importance of assessing nicotine, alcohol, and other substance use, as well as gambling, when considering the success or not of treatment. Contrary to Soyka and colleagues’ (6) study, decreased cocaine use was the one most observed in the “successful OUDT” group. In this case, it is possible that relapse prevention strategies (identification and prevention of high-risk situations to use illicit substances, development of new coping skills, etc.) associated with OUDT also promote the reduction of cocaine use. Possible reasons why cocaine use decreased while alcohol consumption worsened may be related to socioeconomic parameters; compared to cocaine, alcohol is cheaper, more readily available, and more socially acceptable.

Third, our results showed that only 40% of the patients were successfully treated for their OUD, as defined by our study design, despite a long duration of OST and, on a broader level, of global treatment. Nearly three-quarters of the sample were considered as opioid abstinent, but this result is falsely reassuring. Indeed, the main condition explaining the unsuccessful OUDT was not the persistence of opioid use, but the worsening of other substance use or gambling. This is an important novel finding of our study and demonstrates the need to consider a more comprehensive and global outcome measure of a patient’s real life conditions. We chose to use a composite criterion. Notably, expected improvement in the whole addictive situation cannot be directly and solely attributed to OST. From a pharmacological perspective, the primary efficacy of an agonist relies in the decreased consumption of substances acting on the same receptors.

There is no specific consensual criterion used to assess the treatment efficacy. Many studies have explored the follow-up of patients over several years, but with heterogeneous outcome measures and thus heterogeneous results. Regarding the relative effectiveness of OST, Nielsen et al. (11) performed a meta-analysis and used the rates of self-reported opioid use, opioid positive urine drug tests, and retention in treatment as outcomes. They did not find any difference between methadone and buprenorphine maintenance treatment (11). Another meta-analysis by Ma et al. (12) reported an all-cause crude mortality rate of 0.92, 1.69, and 4.89 *per* 100 person-years for those receiving OST, after cessation, and for the untreated period, respectively. In a cohort of 621 people who injected drugs in Tijuana, only 7.3% ceased intravenous drug use (IVDU) at 1 year, but following a methadone maintenance treatment was associated with a significant reduction of IVDU with an odds ratio of 2.04 (1.02–4.08) (15). A recent clinical trial comparing extended-release naltrexone and buprenorphine was based on time to opioid relapse as the outcome measure. It is now completed, but to date the results are not yet available (16). The study by Soyka et al. (6) is similar to ours, with a combination of multiple parameters including unsanctioned opioid abstinence and concomitant drug use. They found an OST retention rate of 70% at 6 years and a mortality rate of 1% *per* year for patients under OST. Other findings from this study were the rates of self-reported opioid use and of opioid positive urine samples at the end of evaluation, respectively, 5% and 12%. These rates were lower than those found in our sample, with 25% patients declaring the persistence of opioid use.

It is possible that our “hard” criterion could partially explain the low rate of successful OUDT. Patients were asked about the evolution of substance use and gambling since the OST initiation, on average 4 years before the inclusion in the study. The design of our study also allowed us to retrospectively analyze those patients who had ceased opioid use following treatment. It was conducive to observe changes, either improvement or worsening, unlike trials with only a 6-month follow-up design (6, 44). Nevertheless, the lack of a consensual definition of OUDT success enforces the need for a global and nonpartial evaluation of other substance use disorders and behavioral addiction.

Finally, we studied factors associated with successful/unsuccessful OUDT. Living as a couple was associated with successful OUDT. The absence of family support has already been described as predictive for unsuccessful OUDT by Hoang et al. (45) and Tran et al. (46, 47). Among all negative consequences, only somatic and financial issues were associated with successful OUDT. They helped the patients to quit opiates but also to improve or stabilize other substance use or gambling. We can assume that these types of negative consequences contribute to make OUD more significant, to limit the switching addiction phenomenon, and to increase intrinsic motivation to a regular medical and social management. On the contrary, factors that lead to substance use (drug-using friends) or to patient isolation (psychiatric and professional negative consequences) are associated with unsuccessful OUDT. Psychiatric issues related to substance use are associated with a lack of insight. OUD can induce psychiatric issues that can in turn promote opioid use, or other substance use and gambling, as a way to cope with negative feelings and emotions. Our patients were recruited in different healthcare centers, but all have a specialization in addiction medicine, so it is possible that patients were more likely to have more severe OUD and more psychiatric comorbidities that can explain the unsuccessful rate. Furthermore, psychiatric comorbidities were already described as factors associated with bad prognosis (6, 48). Professional issues mainly refer to the inability to find and keep a job, which may contribute to patient isolation, to increase boredom and to decrease self-esteem, all these conditions being risk factors for addictive disorders. To avoid these negative consequences, psychosocial treatment aims to modify the underlying processes that maintain or reinforce use behavior, encourage pharmacotherapy compliance, and treat any concomitant psychiatric disorders. It can help patients to manage cravings, reduce the likelihood of relapse, and assist them in coping with the emotional and social challenges (9). Impulsivity, especially high scores for lack of premeditation (tendency to act without thinking) and sensation seeking (tendency to seek out novel and thrilling experiences), was associated with unsuccessful OUDT. Opioid users are known to be impulsive and sensation seekers. Recent findings suggest, on the one hand, impairment in top-down executive control processing and, on the other hand a possible connection between the neuronal reward-signaling system and an excessive need to seek sensory experiences in heroin abusers (49). Other authors concluded that impulsivity, especially lack of premeditation and sensation seeking dimensions, influences the relationship between the desire to abstain from drug use and use in the past month (50). In reference to the Transtheoretical Model of Prochaska and DiClemente, our results confirm that these two facets of impulsivity account for the “intention–action” gap during OUDT. Finally, having more than one OST prescriber was also an important factor associated with unsuccessful OUDT. “Doctor shopping”—the process of seeking multiple prescriptions from multiple prescribers—is a proxy of poor compliance and may also reflect the OST resale on the black market. In France, methadone delivery regulation (51) generally limits this risk of OST misuse, reinforcing the relevance of those restrictive measures.

### Strengths and Weaknesses

Our study has some limitations. First, its design was not longitudinal but cross-sectional, making survival analysis impossible. We overcome this obstacle by determining OUDT success at inclusion and assessing patients’ characteristics partially in a retrospective way. Our results must be interpreted with caution because retrospective data collection over two distinct periods at a unique time can be subject to memorization bias. In addition, the multiplicity of recruiting centers and practitioners means that we cannot provide a participation or refusal rate. Although this was not OPAL’s objective, the only effect of the OST cannot be asserted because of the methodology employed. In particular, the only judgment we can reliably conclude on is the results of the global support considered as successful or not, but not on the evolution through time or the improvement on some clinical characteristic like impulsivity. In this regard, a placebo-controlled randomized clinical trial would have been a way to overcome this methodological bias, which is difficult to conduct given the ethical issues. Second, we deliberately built a semistructured interview to explore OUD characteristics (and especially its negative consequences, for example, psychiatric one), supplemented by validated screening tools or self-rated questionnaires, rather than to use a validated psychiatric interview. We made this decision in order to shorten the assessment visit and to make it more acceptable for the patient. Hence, psychopathological evaluation can be considered as imprecise, and current or past psychiatric disorders were not specified. On the other hand, the patient was questioned about the psychiatric negative consequences of opioid use. No formal diagnosis was made during the interview, but examples such as “depression,” “anxiety,” or “sleep disorders” were proposed as possible consequences for a closer assessment of the patient’s experience. We relied on his/her own perception of the situation when he/she was still using opioid, before the OST initiation. In a large study by Carrà et al. (52), dependence was found to be associated with poorer quality of life and psychosocial adjustment in patients with schizophrenia, and we also know that mentally ill patients are more likely to have severe dependence (53). In light of our data, this means that the installation of a dependence in the presence of a psychiatric comorbidity establishes a vicious circle, making the possibility of therapeutic success even more complicated. Third, we did not perform urine tests to screen substance use in addition to declarative status. We may assume that the unsuccessful OUDT rate could be underestimated.

However, these limits are compensated by the strengths of the study. Despite a single-nation sample, which could limit the representativeness and extrapolation of the results, our recruitment was multicentric with various modalities of practice for services (hospital services, sanitary structures, prison, etc.) that made the population heterogeneous. Heterogeneity of the sample was reached in order to improve the representativeness of real-life OUD patients in treatment. Missing data were marginal, and moreover, the number of patients allowed statistical analyses with a sufficient power. This study was based on a global evaluation of the patients and exhaustive assessment of substance and behavioral addictive disorders at several times: before treatment, since OST initiation and inclusion in the study. Moreover, highlighting predictive factors is useful for the clinicians. Sociodemographic and clinical factors identified in this study are easily collectable for clinicians and may thus be easily monitored in routine care in order to adapt and improve treatment options.

### Perspectives

By identifying factors associated with unsuccessful OUDT, our results provided sociodemographic and clinical features that physicians must be aware of. Also, they should consider these factors of poor prognosis in order to provide the most tailored patient care. OST must be part of a comprehensive treatment program that includes psychological and social interventions, with the aim of helping patients to quit opiates and to avoid switching to an alternative addiction. Enhancing motivation and developing new skills are important, but not sufficient. For some patients, those with low premeditation and high sensation seeking, strengthening executive functioning should be a promising avenue in addition to more conventional strategies. Future research should enhance psychiatric comorbidities in addition to having a most comprehensive evaluation of the patient.

## Members of the OPAL-Group

Pierre Bodenez, Marie Grall-Bronnec, Morgane Guillou-Landréat, Bertrand Le Geay, Isabelle Martineau, Philippe Levassor, Paul Bolo, Jean-Yves Guillet, Xavier Guillery and Corine Dano.

## Data Availability Statement

All datasets generated for this study are included in the manuscript.

## Ethics Statement

The studies involving human participants were reviewed and approved by Groupe Nantais d’Éthique dans le Domaine de la Santé (GNEDS), the Advisory Committee on the Processing of Health Research Informa-tion (CCTIRS) and the Data Protection Commission (CNIL). The patients/participants provided their written informed consent to participate in this study.

## Author Contributions

Conception and design of the study: MG-B, GC-B, JL, and CV-V. Acquisition and analysis of data: MG-B, JC, J-BH, JL, MG-L, CV-V, and the OPAL group (members of the OPAL-Group are PB, MG-B, MG-L, BL, IM, PL, PB, J-YG, XG, and CD). Drafting the manuscript or figures: MG-B, E-JL, GC-B, and CV-V.

## Funding

This study was supported jointly by the Mission Interministérielle de Lutte contre les Drogues et les Conduites Addictives (MILDECA) and the Université Paris 13, as part of the call for research projects “PREVDROG” launched by these two organizations in 2011.

## Conflict of Interest

The authors declare that the research was conducted in the absence of any commercial or financial relationships that could be construed as a potential conflict of interest.
